# Health Seeking Behaviour and Healthcare Utilization in a Rural Cohort of North India

**DOI:** 10.3390/healthcare10050757

**Published:** 2022-04-19

**Authors:** Rajaram Yadav, Kamran Zaman, Ayush Mishra, Mahendra M. Reddy, Prem Shankar, Priyanka Yadav, Kaushik Kumar, Rajni Kant

**Affiliations:** 1 Indian Council of Medical Research—Regional Medical Research Centre Gorakhpur (ICMR-RMRC Gorakhpur), Gorakhpur 273013, India; raja.smile85@gmail.com (R.Y.); mishraa871@gmail.com (A.M.); mahendrabmc@gmail.com (M.M.R.); yadav.priyankyadav@gmail.com (P.Y.); kaushikbhumsw@gmail.com (K.K.); 2 Department of Community Medicine, All India Institute of Medical Sciences (AIIMS Gorakhpur), Gorakhpur 273013, India; prem.pshankar@gmail.com

**Keywords:** formal healthcare, health and demographic surveillance system, health services research, public healthcare, rural health

## Abstract

Background: The healthcare infrastructure of a country determines the health-seeking behaviour of the population. In developing countries such as India, there is a great disparity in the distribution of healthcare institutions across urban and rural areas with disadvantages for people living in rural areas. Objectives: Our objectives were to study the health-seeking behavior and factors associated with the use of formal healthcare among the Gorakhpur Health and Demographic Surveillance System (GHDSS) cohort of North India. Methods: The study was conducted in 28 villages from two rural blocks in the Gorakhpur district of eastern Uttar Pradesh, North India. Structured questionnaires were used to collect the data with regard to demographics, health-seeking behaviour and healthcare utilization. An adjusted odds ratio with 95% confidence interval was used to report the factors associated with the utilization of healthcare. Results: Out of 120,306 individuals surveyed, 19,240 (16%) individuals reported having any health problem in the last 15 days. Of them, 90% sought healthcare for their health needs. The formal healthcare utilization was 79%. The use of public health facilities was very low (37%) with most of the people seeking treatment from private healthcare (63%). Females, people with a higher level of education (graduate and above), and those belonging to rich and middle tercile were more likely to use formal healthcare services. Among different ailments, respiratory problems, gastrointestinal problems, and musculoskeletal problems were associated with decreased use of formal healthcare. Conclusion: About four in five individuals surveyed who had health problems sought treatments from formal healthcare with three in five preferring private institutions to public healthcare facilities due to a perceived higher level of treatment quality and nearby availability. There is an urgent need to re-establish community trust among public healthcare facilities with a focus on delivering on-site health care and enhancing the quality of services offered by public healthcare institutions.

## 1. Introduction

When a person becomes unwell, health-seeking behaviour entails going to a healthcare centre or using a home remedy [1]. The individual’s choice covers all existing healthcare options such as public or private, traditional or modern health care facilities, self-medication, or to not use any health services [2]. Many factors are associated with health-seeking behaviour, namely the type of sickness, degree of illness, gender, surrounding social environment, cost of care, social beliefs about the cause of illness, quality of care, education and economic background [3,4]. A systematic review that analysed health-seeking behaviour concluded that health-seeking behaviour is a multi-dimensional concept and depends on the context and time an individual is facing [5].

The healthcare infrastructure of a country determines the health-seeking behaviour of that country’s population [3,6]. People in most developed countries are covered by universal health coverage (UHC) which is funded by the government. However, in developing countries such as India, UHC is still a distant objective, with out-of-pocket spending accounting for the majority of healthcare costs. Despite significant government investment, convenient access to healthcare remains a major issue. The urban-rural differentials in terms of health infrastructure distribution are very much skewed in India with about 80% of health infrastructure catering to urban India. Rural India, wherein two-thirds of India’s population comes from, is left with very less availability of medical manpower forcing them to utilize the services of traditional healers or go for home remedies or self-medication at large [7,8,9]. 

Similar to health-seeking behaviour, healthcare utilization is influenced by a multitude of factors and is a dynamic concept that is again dependent on time. In general factors such as accessibility, comprehensiveness of care, and continuum of care decides the utilization of healthcare facilities [10]. The utilisation of healthcare facilities in India varies greatly between socioeconomic categories [11]. People in developing countries, such as India, prefer to use private healthcare facilities because they are easier to access and provide more personalised care, whereas public facilities are perceived to be of low quality, located in remote areas, and having long waiting times and insufficient facilities [12,13]. Due to financial constraints, some poor people chose self-treatment or no therapy [14]. 

While there have been studies looking into the health-seeking behaviour of communities in India and other countries, the majority of them have significant limitations. Most of the studies used a smaller sample size and targeted one or more specific strata of populations, such as the elderly, women, and so on [6,7,9,15]. As a result, there is a scarcity of a comprehensive study on this topic with an adequate sample size covering all strata of the population rather than looking at them in sections. Tejas Shah et al. studied the health-seeking behaviour of urban and rural communities in Gujarat’s Ahmedabad district and discovered that healthcare utilisation was significantly lower in the rural area than in the urban area. Although this study provided useful information, it only included 500 households [9]. Similarly, a cross-sectional study of rural women in Telangana discovered that formal healthcare utilisation was lower among rural women. However, the study was limited to three villages and with a sample size of 200 women, limiting the generalizability of the findings. Furthermore, the study only included women and provided no information or comparison of healthcare utilisation by other social groups [15]. 

Our study attempts to overcome these limitations by including a large sample size (~120,000) and including all sections of society. This will enable us to obtain a comprehensive view of the community’s health-care seeking behaviour. Furthermore, because we have already established the Health and Demographic Surveillance System (HDSS), the community’s health care seeking behaviour can be studied over time, unlike previous studies which were limited to a single point of time. Due to all of these factors, our study’s findings may be more relatable to existing times wherein government interventions are targeted to improve universal health coverage. The present study was carried out to investigate the factors associated with health-seeking behaviour and healthcare utilization in the cohort of the Gorakhpur Health and Demographic Surveillance System (GHDSS). 

## 2. Material and Methods

This study was conducted in the rural cohort of the GHDSS site, which includes 28 villages from 2 blocks (Chargawa and Bhathat) in the Gorakhpur district, Uttar Pradesh. The site includes a total of 20,965 households with a population of about 120,306 people. The baseline data were collected from November 2019 to February 2021. The enumeration survey included all individuals in the study area except those who declined to participate or whose doors were locked. If a door was found to be locked during the initial visit, the household was revisited, and if two additional attempts failed, the house was reported as locked. 

Before beginning the interview, informed verbal consent was obtained from the head of the family and, if he/she was unavailable, from the available elder member of the household. Preferably, the head of the household (HoH) was questioned, but if the HoH was not present, any member of the household over the age of 18 was interviewed for pretested open data kit (ODK) based questionnaires to collect data on the household’s health and demography. Field investigators were supervised and overseen by field supervisors and project scientists while they collected the data. 

The wealth tercile was determined in STATA using principal component analysis based on the existence or absence of specific assets in the household. After dividing the population into five quintiles, the first and second quintiles were merged to form the poor tercile, the fourth and fifth wealth quintiles were merged to form the rich tercile, and the third quintile was designated as the middle tercile.

The utilization of the healthcare facilities was operationally categorized into either ‘formal’ or ‘informal’ healthcare. Formal healthcare included receiving treatment from both public and private health care providers and Informal healthcare included receiving treatment from traditional healers and by self-medication. Public health facilities included health care facilities of the government (state/central) which provided health care facilities free of cost or a nominal/subsidised rate and the private health facility included health care facilities other than those provided by the government and includes private hospitals and private clinics.

In this study, data from only those persons who had any health problem in the last 15 days preceding the survey were analysed, allowing healthcare used to be estimated among the population in need of care. First, the data were descriptively analysed based on selected household and individual variables. For regression analysis, using formal healthcare (yes/no) was taken as the dependent variable and all categorical variables including household size category, gender, relation to household head, education, marital status, occupation, religion, wealth tercile, age groups, caste and ailments (infection, cancer, blood disease, endocrine metabolic and nutrition, psychiatric and neurological, eye disease, ear disease, cardiovascular disease, respiratory infection, ad gastrointestinal disease, musculoskeletal, obstetric, and injuries) were used as independent variables. Binary logistic regression was carried out to determine the factors associated with the utilization of formal healthcare. All factors were included in the final multivariable logistic regression model and the association was reported using adjusted odds ratio (OR) along with a 95% confidence interval (CI). The model significance was reported using Nagelkerke’s pseudo R^2^ and model *p*-value [16]. A *p*-value of less than 0.05 was considered to be a statistically significant association. All analyses were carried out using STATA-14 software (StataCorp LP, College Station, TX, USA).

## 3. Results

Of the total 120,306 population surveyed, males accounted for 51.9% of the population in the study, and 7.9% of the population were 60 years or older. The literacy rate in the study area was 73.7%, with around 7.1% of the population having an education level of graduation and above. Approximately 38.1% of the population was in the rich tercile and 29.2% in the poor tercile. Table 1 presents the complete demographic details of the study population.

**Figure 1:**
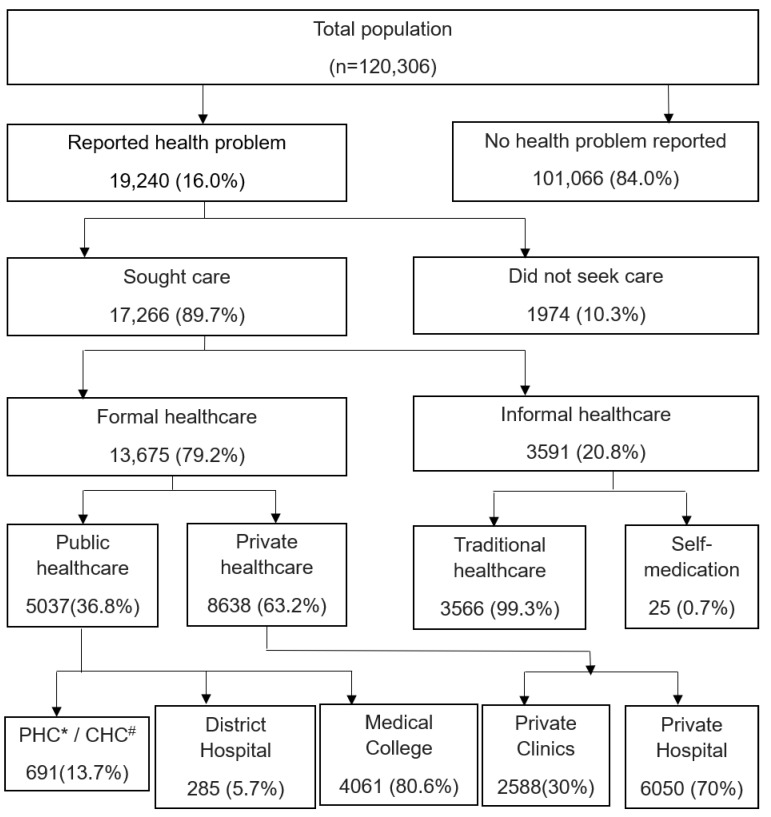
Flow diagram depicting health-seeking behaviour and healthcare utilization among people of GHDSS cohort (*n* = 120,306). * PHC, primary health centre; ^#^ CHC, community health centre.

A total of 19,240 (16%) people reported some form of illness/disease in the last 15 days prior to the survey. Of them, any healthcare facility was used by 89.7%, while 10.3% did not seek treatment at all. The use of public healthcare facilities was noted to be low (36.8%) when compared to private healthcare facilities (63.2%). Among them, 20.8% of individuals seeking informal healthcare, traditional healers were approached the maximum (99.3%) followed by self-medication in 0.7% of individuals (Figure 1). 

**Figure 2:**
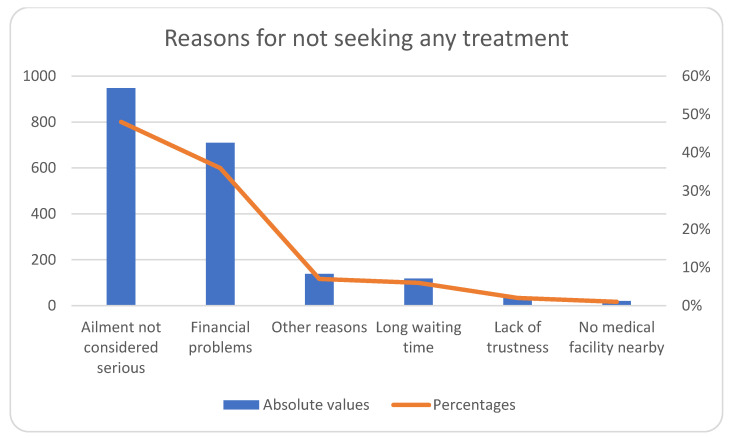
Reasons for non-utilization of healthcare services among people who reported any ailment in the GHDSS cohort (*n* = 1974).

The most often reported reasons for not seeking treatment by study participants were that of ailment being ‘not considered to be serious’ (48%) followed by ‘financial constraints’ (36%) (Figure 2).

Among the various diseases, obstetric problems had the lowest rate of medical treatment utilisation, with only 76% of the respondents receiving medical treatment, while cardiovascular diseases and endocrine and metabolic diseases had the highest rate, with 98% receiving medical treatment (Table 2).

Among those who sought treatment, 21.4% (3711) reported changing in medical consultation after the first visit. Most of them (around 90%) reported no relief as a reason for doing so and 4.9% reported financial problems as the reason for changing medical consultation. No significant difference between private (48.4%) and public healthcare (51.6%) was observed in terms of change of the first consultation due to no relief.

Table 3 shows the variation in usage of formal healthcare and informal healthcare in relation to demographic characteristics. People living with a family size of six or more were 1.10 times higher odds of utilizing formal healthcare services for their treatment than those with a family size of five or less. In terms of gender, males had 1.21 times higher odds than females to use formal healthcare. When it comes to intra-household relationships; children (1.34 times), the spouse of children (1.47 times), and grandchildren (1.46 times) are more likely, while parents (0.80 times) are less likely to use formal healthcare compared to the head of the household.

People having education level up to higher secondary (1.19 times) and people having education level of graduation and above (1.76 times) are more likely to use formal healthcare as compared to those who are illiterate. People with regular wage/salary are around 2.3 times (*p* < 0.001) more likely to use formal healthcare as compared to people self-employed in agriculture. When compared to the poor, the middle group is 1.51 times (*p* < 0.001) and the rich are 2.19 times (*p* < 0.001) more likely to use formal healthcare. People in the age groups 15–29, 30–59, and 60 years and above have a, respectively, 1.35 times, 1.90 times, and 1.81 times higher odds of using formal healthcare as compared to people in the age group 0–14 years. 

With respect to ailments, after adjusting to all variables except for injuries and skin ailments all other ailments had significant association with usage of formal healthcare. Among infections, respiratory problems, gastrointestinal problems, and musculoskeletal problems were associated with decreased use of formal healthcare (see Table 3).

## 4. Discussion

The under-utilization of a public healthcare facility is common in all developing countries whereas the use of private healthcare is growing in developing and under-developed countries [2]. It is found in our study that most of the population prefers to use private healthcare facilities viz. private hospitals, private doctors, or private clinics. A similar finding was observed in a previous study which was carried out in Bangladesh [17]. Private facilities are preferred since they are available nearby and are believed to have a better quality of care [2]. People have a prevalent belief that private healthcare institutions give superior care to public healthcare facilities [18]. 

We found in our study that there is a significant gender difference in the utilization of formal healthcare, wherein it was found that utilization of formal healthcare services was higher among males as compared to females, which is contradictory to the finding of another study previously carried out in India [11]. The differences could be due to the higher prevalence of the patriarchal system in this part of the country compared to the study from northeast India. We did not find any significant association between religion and the utilization of formal healthcare in this study. This may be due to the low distribution of other religions apart from Hindus in our study population. 

It was also observed in our study that people having higher education (higher secondary and graduate and above) are more likely to use formal healthcare since they are more aware of their health. In the case of the relationship between marital status and formal healthcare utilization, it is found in this study that widows are significantly less likely to use formal healthcare for their treatment as compared to those who never married. This is also supported by other previous studies [19,20]. 

We also found that people who belong to rich or middle-class families are significantly more likely to seek treatment from formal healthcare as compared to the poor, which is also evident from the study carried out in Bangladesh [17]. The reasons for non-utilization could be due to their disadvantaged status in the community making them have poor awareness, access, and beliefs in the healthcare system. 

People with higher age categories (above 14 years) used the formal healthcare system more compared to those in the 0–14 years category. This may be due to the fact that the decision-making process in this age group is in the hands of caregivers who may be influenced by social beliefs. 

We found in our study that people seek the help of traditional healers or informal healthcare for diseases such as musculoskeletal diseases, fever of unknown origin (18%), and upper respiratory tract diseases. The major cause of this trend is that people do not consider these diseases as serious. In addition, traditional therapy is considered to be harmless. Similar findings were seen in previously conducted studies in Sierra Leone and Indonesia [21,22]. In our study, we have not captured the severity of disease, which may be one of the important factors to decide in seeking for healthcare.

Further, we found that household size was independently associated with the usage of formal healthcare. Larger household sizes (six and above) compared to lesser household sizes (five and below) have higher odds of using formal healthcare. The reason for this needs to be further explored. In our study, we also found that the relationship with the head of the household also determines the usage of formal healthcare. Children of the head, spouse of the children, and grandchildren use formal healthcare more compared to the head of the household. Also, compared to the head of the household, parents of the head are using formal healthcare significantly lesser. This may be due to the beliefs and also the perceived status of the head of the household, who generally decides the usage of healthcare (especially in rural areas). This could also be attributed by the changing healthcare seeking behaviour across generations with younger generation making informed decisions based on better awareness compared to elderly.

The study findings may be generalizable to similar settings across India and also other lower-middle-income countries. The study has few implications. The study calls for more focus on health infrastructure in rural India and also increased awareness to improve health-seeking behaviours and healthcare utilization across rural India. The study also calls for health insurance coverage for people living in rural India which may bring about a change in health-seeking behaviours and health care utilization by reducing out-of-pocket health expenditures. Furthermore, ailments such as respiratory diseases are having lesser usage of formal healthcare which could have huge consequences in terms of morbidity and mortality especially in paediatric age groups. This calls for increased awareness in rural areas through existing maternal and child health programmes in seeking for formal healthcare in case of ailments such as respiratory infections which may have a huge bearing on outcome if there is a delay in seeking formal healthcare systems. Also, with respect to neglect of seeking healthcare among adults, respiratory infections may derail in achieving the goal of ending tuberculosis (TB) by 2025 in India. There is also a need to increase the formal healthcare system (especially the public health care system) so that it is more accessible and also reduce the health expenditures in rural India. With the advent of Ayushman Bharat and Health and wellness centres in India, the solution for removing the skewness in health coverage across rural and urban India may well be on cards [23]. Health-seeking behaviour and healthcare utilization must be one of the prominent indicators, especially in rural India to assess the implementation of such schemes in future. 

## 5. Strengths and Limitations of the Study

The study’s strength is that it is based on complete enumeration. Therefore, there is no sampling bias in the study. The study is a part of large cohort with ~120,000 population which makes the findings to be more generalizable. By limiting the morbidity reference period to 15 days before the survey, the utmost effort was taken to reduce recall bias. We have adjusted the analysis so that the independent factors associated with formal healthcare use are determined more accurately. One significant drawback of the study is that morbidity and health-seeking behaviour are quantified based on reported sickness and treatment received rather than being observed or diagnosed. As a result, there is a chance of under-reporting of diseases for which formal care was not sought. Further, as mentioned earlier we have not captured the severity of disease which could be an important factor in seeking for healthcare. Also, the availability of formal healthcare is another factor in deciding the usage of formal healthcare. A variable such as the nearest distance from a particular household to the formal healthcare facility (private/public) would have bought more insights in health-seeking behaviours. 

## 6. Conclusions

This study provides rich information about the local community’s health-seeking behaviour. Although 80% seek formal healthcare for their ailments, three in five persons who sought care preferred private institutions to public healthcare facilities due to a perceived higher level of treatment quality and nearby availability. In our study we found that formal healthcare utilization was significantly higher among males, people having better socioeconomic status and higher age groups (14 years and above). Among different ailments infections, respiratory problems, gastrointestinal problems and musculoskeletal problems were associated with decreased use of formal healthcare. These findings give critical feedback for the development and implementation of healthcare policies. Public healthcare facilities should be extended to underserved areas, with a focus on delivering on-site health care through wellness centres with the assistance of an accredited social health activist (ASHA) and auxiliary nurse midwife (ANM). In order to re-establish community trust in public healthcare facilities, and emphasis should be placed on enhancing the quality of services offered by public healthcare institutions.

## Figures and Tables

**Table 1:** Socio-demographic profile of the population in the study area (*n* = 120,306).

Background Characteristics	Percentage Distribution	
**Gender**		
Male	51.9	
Female	48.0	
Transgender	0.1	
**Age groups**		
0–14	30.0	
15–29	31.7	
30–59	30.4	
60+	7.9	
**Dependency ratio**	49.3	
**Education level**		
Illiterate	26.3	
Literate without schooling	2.6	
Below primary	11.6	
Primary	13.4	
Middle	17.1	
Secondary	10.9	
Higher secondary	10.8	
Graduation and above	7.1	
**Marital status among adult population**		
Never married	22.0	
Currently married	71.1	
Divorced/separated	0.3	
Widowed	6.6	
**Occupation**	**Females**	**Males**
Self-employed in agriculture	0.8	14.5
Self-employed in non-agriculture	0.2	7.9
Regular wage/salary earning	0.6	5.5
Casual labor	1.0	49.9
Housewife	59.1	-
Student	34.4	15.8
Others	3.9	6.4
**Religion**		
Hindu	94.7	
Minority	5.3	
**Tercile groups**		
Poor	29.2	
Middle	32.7	
Rich	38.1	

**Table 2:** Health-seeking behaviour across various diseases and their sub-analysis in formal and informal healthcare.

Type of Disease (*n*)	Sought Healthcare *n* (%)	Formal Healthcare *n* (%)	Informal Healthcare *n* (%)
Infection (1659)	1567 (94.4)	991 (63.2)	576 (36.8)
Cancers (313)	275 (87.9)	255 (92.7)	20 (7.3)
Blood disease (37)	33 (89.2)	32 (97.0)	1 (3.0)
Endocrine, metabolic and nutrition (1670)	1632 (97.7)	1547 (94.8)	85 (5.2)
Psychiatric and neurological (1821)	1602 (88)	1474 (92.0)	128 (8.0)
Eye (729)	596 (81.8)	538 (90.3)	58 (9.7)
Ear (638)	546 (85.6)	465 (85.2)	81 (14.8)
Cardio vascular diseases (1688)	1648 (97.6)	1499 (90.1)	149 (9.0)
Respiratory (2000)	1835 (91.7)	1325 (72.2)	510 (27.8)
Gastro intestinal (1319)	1205 (91.4)	902 (74.9)	303 (25.1)
Skin (969)	894 (92.3)	689 (77.1)	205 (22.9)
Musculoskeletal (3619)	3313 (91.5)	2321 (70.1)	992 (29.9)
Genitourinary (1563)	1399 (89.5)	1189 (85.0)	210 (15.0)
Obstetric (338)	258 (76.3)	251 (97.3)	7 (2.7)
Injuries (4283)	3710 (86.6)	2935 (79.1)	775 (20.9)

**Table 3:** Socio-demographic factors associated with usage of formal healthcare among people living in the GHDSS cohort (*n* = 17,266).

Characteristics	*n* = 17,266	Usage of Formal Healthcare, *n* (%)	Unadjusted OR ^@^ with 95% CI		Adjusted OR ^#^ with 95% CI	
**Household size**						
1–5	7125	5500 (77)	1		1	
6 and above	10,141	8175 (81)	1.23 ***	[1.14, 1.32]	1.10 *	[1.00, 1.21]
**Gender**						
Female	9519	7378 (78)	1		1	
Male	7733	6286 (81)	0.79 ***	[0.74, 0.86]	1.21 *	[1.05, 1.41]
Transgender	14	11 (79)	0.84	[0.23, 3.03]	0.94	[0.25, 3.56]
**Relation to the head of household**						
Self	5106	4091 (80)	1		1	
Spouse	4930	3837 (78)	0.87 **	[0.79, 0.96]	1.00	[0.84, 1.0]
Child (Son/daughter)	4107	3271 (80)	0.97	[0.88, 1.08]	1.34 **	[1.08, 1.66]
Spouse of child	916	780 (85)	1.42 ***	[1.17, 1.73]	1.47 **	[1.13, 1.91]
Grand child	835	636 (76)	0.79 **	[0.67, 0.94]	1.46 *	[1.04, 2.03]
Father/Mother	1054	790 (75)	0.74 ***	[0.64, 0.87]	0.80 *	[0.66, 0.96]
Brother/Sister	191	153 (80)	1.00	[0.70, 1.43]	0.77	[0.51, 1.16]
Other relative	122	113 (93)	3.12 **	[1.57, 6.16]	3.98 ***	[1.88, 8.45]
Not relative	5	4 (80)	0.99	[0.11, 8.89]	1.70	[0.16, 18.31]
**Education**						
Illiterate	7451	5682 (76)	1		1	
Up to higher secondary	8300	6763 (81)	1.37 ***	[1.27, 1.48]	1.19 **	[1.07, 1.32]
Graduation and above	830	759 (91)	3.33 ***	[2.59, 4.27]	1.79 ***	[1.35, 2.36]
**Marital status**						
Never married	3538	2806 (79)	1		1	
Currently married	11,056	8883 (80)	1.07	[0.97, 1.17]	0.81	[0.63, 1.04]
Divorced/separated	61	54 (88)	2.01	[0.91, 4.44]	1.19	[0.51, 2.77]
Widowed	1926	1456 (76)	0.82 **	[0.72, 0.94]	0.76 *	[0.57, 1.01]
**Occupation**						
Self-employed in agriculture	1699	1383 (81)	1		1	
Self-employed in non-agriculture	573	483 (84)	1.23	[0.95, 1.58]	0.87	[0.66, 1.14]
Regular wage/salary	459	431 (94)	3.52 ***	[2.35, 5.25]	2.08 ***	[1.37, 3.16]
Casual labour	2791	2196 (79)	0.84 *	[0.72, 0.98]	0.93	[0.79, 1.10]
Housewife	7158	5594 (78)	0.82 **	[0.71, 0.93]	1.04	[0.84, 1.29]
Student	2369	1821 (77)	0.76 ***	[0.65, 0.89]	0.82	[0.63, 1.07]
Others	1532	1296 (85)	1.25 *	[1.04, 1.51]	1.35 **	[1.09, 1.68]
**Religion**						
Hindu	16,189	12,800 (79)	1		1	
Muslim	1033	839 (81)	1.15	[0.98, 1.34]	1.05	[0.88, 1.27]
Others	3	3 (100)	1.00	[1.00, 1.00]	1.00	[1.00, 1.00]
**Wealth tercile**						
Poor	5321	3744 (70)	1		1	
Middle	5545	4383 (79)	1.59 ***	[1.46, 1.73]	1.51 ***	[1.37, 1.66]
Rich	6359	5515 (87)	2.75 ***	[2.51, 3.02]	2.19 ***	[1.96, 2.44]
**Age groups**						
0–14	2173	1575 (72)	1		1	
15–29	3291	2669 (81)	1.63 ***	[1.43, 1.85]	1.35 **	[1.11, 1.64]
30–59	7867	6356 (81)	1.60 ***	[1.43, 1.78]	1.90 ***	[1.48, 2.44]
60+	3935	3075 (78)	1.36 ***	[1.20, 1.53]	1.81 ***	[1.38, 2.37]
**Caste**						
Scheduled Caste/Scheduled Tribe	5080	3869 (76)	1		1	
Other Backward Class	11,232	8975 (80)	1.24 ***	[1.15, 1.35]	1.07	[0.98, 1.17]
Others	913	798 (87)	2.17 ***	[1.77, 2.67]	1.20	[0.95, 1.51]
**Type of ailments**						
**Infection**						
No	15,704	12,700 (81)	1		1	
Yes	1562	975 (62)	0.39 ***	[0.35, 0.43]	0.46 ***	[0.39, 0.53]
**Cancers**						
No	16,991	13,420 (79)	1		1	
Yes	275	255 (93)	3.39 ***	[2.15, 5.36]	3.18 ***	[1.98, 5.09]
**Blood disease**						
No	17,233	13,643 (79)	1		1	
Yes	33	32 (97)	8.42 *	[1.15, 61.64]	8.46 *	[1.14, 62.99]
**Endocrine, metabolic and nutrition**						
No	15,640	12,134 (78)	1		1	
Yes	1626	1541 (95)	5.24 ***	[4.20, 6.54]	3.53 ***	[2.78, 4.49]
**Psychiatric and neurological**						
No	15,670	12,206 (78)	1		1	
Yes	1596	1469 (92)	3.28 ***	[2.73, 3.95]	2.76 ***	[2.23, 3.41]
**Eye diseases**						
No	16,671	13,138 (79)	1		1	
Yes	595	537 (90)	2.49 ***	[1.89, 3.27]	2.61 ***	[1.96, 3.48]
**Ear diseases**						
No	16,724	13,214 (79)	1		1	
Yes	542	461 (85)	1.51 ***	[1.19, 1.92]	1.68 ***	[1.28, 2.20]
**Cardio vascular diseases**						
No	15,623	12,183 (79)	1		1	
Yes	1643	1492 (91)	2.79 ***	[2.35, 3.31]	2.07 ***	[1.71, 2.50]
**Respiratory**						
No	15,444	12,364 (80)	1		1	
Yes	1822	1311 (72)	0.64 ***	[0.57, 0.71]	0.74 ***	[0.63, 0.86]
**Gastro intestinal**						
No	16,064	12,776 (80)	1		1	
Yes	1202	899 (75)	0.76 ***	[0.67, 0.87]	0.72 ***	[0.61, 0.85]
**Skin**						
No	16,379	12,995 (79)	1		1	
Yes	887	680 (77)	0.85	[0.73, 1.00]	0.88	[0.72, 1.07]
**Musculoskeletal**						
No	13,981	11,388 (81)	1		1	
Yes	3285	2287 (70)	0.52 ***	[0.47, 0.57]	0.57 ***	[0.50, 0.66]
**Genitourinary**						
No	15,878	12,498 (79)	1		1	
Yes	1388	1177 (85)	1.51 ***	[1.30, 1.76]	1.37 ***	[1.14, 1.64]
**Obstetric**						
No	17,009	13,425 (79)	1		1	
Yes	257	250 (97)	9.53 ***	[4.50, 20.22]	7.96 ***	[3.70, 17.12]
**Injuries**						
No	13,569	10,757 (79)	1		1	
Yes	3697	2918 (79)	1.49 ***	[1.45, 1.53]	1.02	[0.89, 1.17]

* *p* < 0.05, ** *p* < 0.01, *** *p* < 0.001, ^@^ Unadjusted Odds Ratio, ^#^ Adjusted Odds Ratio and model significance: pseudo R^2^ = 0.0984, *p* value < 0.001 (*n* = 16,541).

## Data Availability

The datasets used and/or analysed during the present study are available from the corresponding author on reasonable request.
